# A Meta‐Synthesis Exploring Daily Experiences of Adults With Coeliac Disease in Adhering to a Gluten‐Free Diet

**DOI:** 10.1111/jhn.70043

**Published:** 2025-04-08

**Authors:** Anna Kowalczuk, Fiona Moor

**Affiliations:** ^1^ Faculty of Health and Life Sciences Coventry University Coventry UK

**Keywords:** coeliac disease/celiac disease, experiences, gluten‐free diet, meta‐synthesis, qualitative

## Abstract

**Background:**

Coeliac disease (CD) is an autoimmune disease affecting 1.4% of the population worldwide. The only treatment for this condition is a strict, lifelong gluten‐free diet (GFD). Although the complexity of this condition is recognised, the definitive follow‐up strategy and long‐term management have still not been developed in the United Kingdom (UK) and Australia. This meta‐synthesis aimed to explore the experiences of patients living with CD who follow the GFD in the UK and Australia.

**Methods:**

A systematic search for primary qualitative literature related to experiences of patients with CD on a GFD and a meta‐synthesis of the results were conducted. Healthcare‐relevant online databases were screened: Academic Search Complete, CINAHL, MEDLINE, and Scopus, followed by reference list searching. A defined inclusion criteria were used to identify relevant studies. The data synthesis from the literature followed the thematic synthesis approach. A clear description of the methodology and peer review were applied to ensure transparency.

**Results:**

A total of 286 studies were screened for eligibility. Of those, six studies met the inclusion criteria. The experiences of 198 patients living with CD were analysed and reported. Five analytical themes emerged through the thematic synthesis process: acceptance and adaptation, dietary burden, cost burden, socialising, and importance of support. All themes were found to impact patients' quality of life (QoL). The overall quality of the reviewed studies was described as good.

**Conclusion:**

This meta‐synthesis revealed insights into the daily experiences of patients with CD in adhering to a GFD in the UK and Australia, being the first secondary qualitative study to explore this phenomenon. It highlighted the need for the development of defined strategies for patient follow‐up to provide holistic care, considering the complexities of this condition and its impact on both physical and psychological domains.

## Summary

- Following a strict gluten‐free diet presents specific challenges, affecting the quality of life of patients with coeliac disease.
- A management strategy for individuals with coeliac disease should involve a multidisciplinary, person‐centred approach.

## 1 Introduction

Coeliac disease (CD) is an autoimmune condition driven by gluten hyperreactivity, which results in atrophy of the intestinal villi [1]. The global prevalence is estimated to be 1.4%, with a 0.2% rise over the last three decades due to increased awareness among physicians, improved diagnostic criteria, and environmental and dietary changes [2]. CD can affect people of any age and sex; however, women are 2.8 times more likely than men to be affected [3, 4]. According to the National Institute for Health and Care Excellence (NICE), 1% of the population in the United Kingdom (UK) is affected [5]. In Australia, the estimates are 1.2% for men and 1.9% for women [6]. However, because of inadequate data from nations and high rates of undiagnosed individuals, the overall figure may be greater [7]. The sole treatment for patients who have CD is lifelong adherence to a gluten‐free diet (GFD), which reduces symptoms and often results in intestinal mucosa regeneration [8]. Many associated health problems in the long‐term management of CD are a consequence of poor dietary adherence, yet still, no definitive monitoring method for patients has been developed in both the UK and Australia [5, 9].

Literature from previous years suggests a strong correlation between CD, GFD adherence, and patients' QoL. [10, 11, 12] Most researchers conduct quantitative studies focusing on the overall experience of patients living with CD and their quality of life (QoL) rather than examining the impact of GFD adherence [13, 14]. Existing primary qualitative research exploring the experiences of people with CD predominantly originates from European countries such as Sweden and Greece, while evidence in the UK and Australia remains limited [15, 16].

Möller et al. [14] found that QoL is closely related to psychological distress, illness beliefs, coping, and attitudes toward food and a GFD. The results revealed that psychological well‐being has a greater impact on QoL than dietary adherence, disease state, or symptoms. Similar issues were explored in a UK‐based narrative review by White, Bannerman, and Gillett [13], with a particular focus on the adolescent population with CD. Unlike Möller et al. [14], they found a strong correlation between GFD adherence and health‐related quality of life (HRQoL).

Adherence to the GFD has been found to have a positive impact on short‐ and long‐term physical health outcomes, but the highly restrictive nature of the diet imposed a significant burden on patients, negatively impacting their daily lives [17]. A systematic review by Haines, Anderson, and Gibson [18] analysed the long‐term management of CD along with its complications. The risk of complications appeared to be multifactorial, depending on environmental, genetic, and dietary factors. They found that strict GFD compliance reduced the inflammation of intestinal mucosa, preventing later health complications such as osteoporosis, malnutrition, and anxiety. This review highlighted the importance of appropriate long‐term management strategies and improved risk assessment methods to minimise the incidence of complications.

Similar conclusions regarding long‐term CD management were drawn in a recent narrative review [19]. The study reviewed and evaluated the various follow‐up strategies available to patients with CD in the UK. It highlighted the challenges in the standardisation process of CD treatments, mainly in the regional dependability of services provided by the National Healthcare Service (NHS). The divided nature of the NHS, with local commissioning groups having the autonomy to decide on the availability of local services, has led to the reduction or removal of support for patients living with CD in 27% of all local service providers, causing regional inequalities in care [20].

The British Society of Gastroenterology recommends that patients who have CD should be offered a dietetic consultation after diagnosis, followed by two reviews at 3 and 6 months, and then an annual follow‐up by a dietitian and treating physician [8]. However, the literature suggests that access to dietetic services for patients living with CD is often limited [21, 22, 23]. In Australia, the management of patients with CD is similar, with the same timeframes for follow‐up appointments; however, the patients are not offered prescriptions for basic GF foods as in some areas of the UK [24, 25].

Gaining insights into patients' experiences could inform the development of an effective model of care for this patient group tailored to health services in the UK and Australia. As the NHS [26] emphasises, the development of new models of care should be patient‐led, based on patients' needs and choices. Additionally, the Health and Care Professions Council (HCPC) [27] outlines the importance of a person‐centred approach taken by a multidisciplinary team.

This study aimed to investigate the experiences of patients with CD on a GFD, to point to directions in which care for this patient group should be focused, and to enhance the qualitative evidence base to support healthcare professionals in adopting a holistic, patient‐centred approach.

## 2 Methods

### 2.1 Methodology/Design

As the study aimed to explore the experiences of patients with CD who adhere to a GFD, it resonated well with following a qualitative approach, allowing an in‐depth analysis of the phenomenon rather than obtaining numerical results [28]. A meta‐synthesis, being secondary research, enables one to gain a broad perspective and develop a greater understanding of the problem by combining the findings of different studies [29, 30]. The project obtained Coventry University Ethics Committee approval (reference number P146307).

### 2.2 Search Strategy and Study Selection

The initial scoping search was conducted in March 2023 to assess the body of evidence [31]. The search through the PROSPERO database [32] ensured there is no similar research in progress. The systematic literature search was performed in June 2023 by the lead researcher and updated in May 2024 by the University Hospitals Birmingham NHS Foundation Trust Library and Knowledge Services to identify any relevant newly published studies. The searches were conducted in online databases applicable to healthcare research: CINAHL, MEDLINE, Academic Search Complete, and Scopus (see Supporting Information S1: Table S4). Reference list searching was an additional search strategy applied to maximise the identification of relevant studies [33].

The search terms were identified by the PEO framework: P—population (patients with CD), E—exposure (GFD), and O—outcome (experience) (see Supporting Information S1: Table S1).

The identification of the inclusion and exclusion criteria ensured consistency within the process of study selection (see Supporting Information S1: Table S2) [34]. Included studies were qualitative and written in English, were based in the UK or Australia, and provided insights into experiences of adult patients having CD who follow a GFD. This meta‐synthesis focused on the UK and Australia due to comparable healthcare systems, similar CD management frameworks, and a lack of secondary qualitative studies examining the experiences of patients with CD on a GF diet in these countries [21, 22, 23, 24, 25].

To ensure transparency, the reasons for the studies' exclusion were made based on the title, abstract, or full‐text assessment. Additionally, the identified studies were discussed with the second researcher to ensure their applicability to the research.

Included studies were appraised using the piloted CASP Tool for qualitative research [35] to identify their strengths and limitations [34]. A specific level of quality was not an inclusion criterion as the evidence on the topic was limited, and lower‐quality studies could still provide relevant insights if their limitations were acknowledged [36].

**Figure 1:**
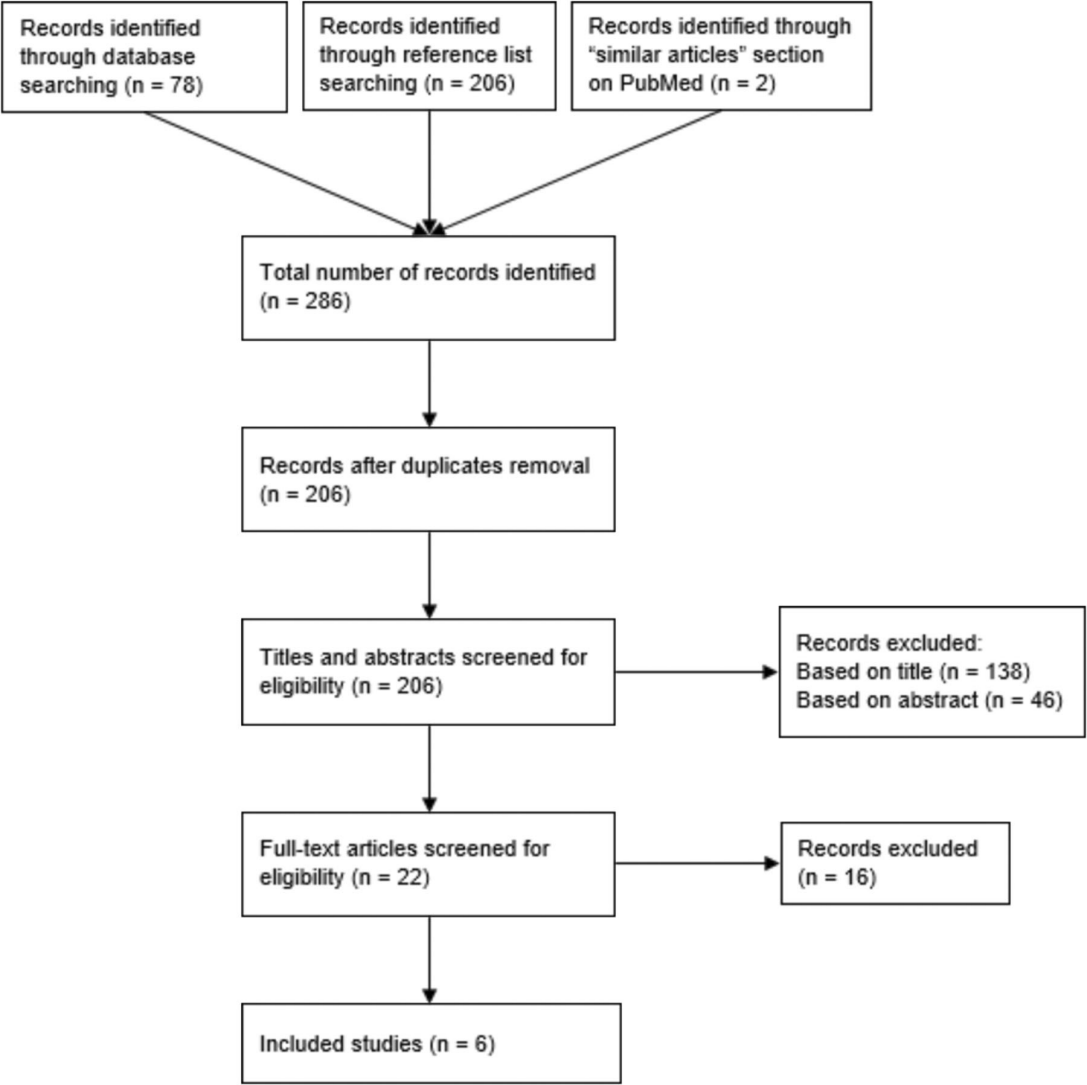
Adapted PRISMA flow diagram.

To illustrate the study selection process, the Preferred Reporting Items for Systematic Reviews and Meta‐Analyses (PRISMA) Flow Diagram [37] and study characteristics are presented in Figure 1.

### 2.3 Data Extraction, Synthesis and Quality Assessment

The data was extracted by the lead researcher using the JBI QARI Qualitative data extraction tool together with the extraction of the findings sheet [38]. Both tools were piloted using one qualitative study, following the PIRSMA checklist [37].

Data analysis of 6 identified studies, performed by the lead researcher, followed the process of thematic synthesis designed for qualitative reviews [39]. This process involved three stages: Line‐by‐line coding of the studies' results, grouping the “free codes” into “descriptive categories” according to their similarities and differences, and creating “analytical themes” from the “descriptive themes”.

Line‐by‐line coding produced 357 “free codes,” which were grouped into 35 descriptive categories and then into 16 descriptive themes. Based on those, five final “analytical themes” were generated following a peer review by the second researcher (see Supporting Information S1: Figure S1).

## 3 Results

### 3.1 Study Characteristics and Sample Demographics

In total, six studies were included, involving the experiences of 198 patients with CD. Characteristics and sample demographics of included studies are presented in Table 1. The age range of participants was broad: 18–78 years. The participants were mostly women, which reflects well the population affected by CD [4]. The time since diagnosis varied from 3 months to 43 years, and only two researchers determined the duration of GFD adherence in participants. Four of the included studies were based in the UK and two in Australia to allow the richness of experiences in comparable healthcare systems.

**Table 1:** Study characteristics and sample demographics.

Year	Authors	Title of study	Country	Study approach	Data collection methods	Method of data analysis	Number and gender of participants	Age range of participants (years)	Time since CD diagnosis	Time on a GF diet
2012	Taylor, Dickson‐Swift & Anderson [40]	Coeliac disease: the path to diagnosis and the reality of living with the disease.	Australia	Qualitative	Face‐to‐face or phone interviews	Not specified	Total: 10 Females: 10	31–60	2–26 years	2–26 years

2013	Rose & Howard [41]	Living with coeliac disease: a grounded theory study.	United Kingdom	Qualitative	Written narratives	Grounded theory	Total: 130 Males: 29 Females: 69 Unspecified: 6	19–78	3 months to 43.1 years	Not specified
2017	Satherley, Higgs & Howard [42]	Disordered eating patterns in coeliac disease: a framework analysis.	United Kingdom	Qualitative	Face‐to‐face interviews	Framework analysis	Total: 21 Males: 5 Females: 16	19–59	2–19 years	Not specified
2019	Peters, Crocker, Jenkinson & Violato [25]	Withdrawing gluten‐free food from prescriptions in England: a mixed‐methods study to examine the impact of policy changes on quality of life.	United Kingdom	Mixed methods	Cross‐sectional survey and face‐to‐face or phone interviews	Not specified	Questionnaire: Total: 1697 Males: 229 Females: 576 Interviews: Total: 24 Males: 11 Females: 13	18–70+	Mean: 13.9 years for all participants Interviewed participants: < 1 year to > 20 years	Not specified
2021	Lee, Crowley, Baines, Heaney & Brown [43]	Patient Perspectives of Living with Coeliac Disease and Accessing Dietetic Services in Rural Australia: A Qualitative Study.	Australia	Qualitative	Phone interviews	Not specified	Total: 6 Males: 3 Females: 3	38–71	< 1–10 years	Not specified
2022	Satherley, Lerigo, Higgs & Howard [44]	An interpretative phenomenological analysis of the development and maintenance of gluten‐related distress and unhelpful eating and lifestyle patterns in coeliac disease.	United Kingdom	Qualitative	Online focus group	Phenomenology	Total: 7 Males: 1 Females: 6	25–49	3–13 years	3–13 years

Abbreviations: CD, coeliac disease; GFD, gluten‐free diet.

### 3.2 Quality Statement

The overall quality of the studies assessed using the CASP Tool [35] was rated as good, and the results are perceived as credible (see Supporting Information S1: Table S3).

### 3.3 Analytical Themes

**Figure 2:**
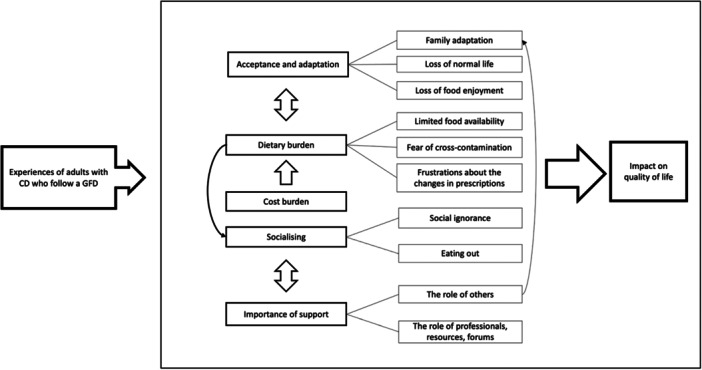
Concept map. CD, coeliac disease; GFD, gluten‐free diet.

Figure 2 presents five identified analytical themes and sub‐themes with mutual relations, describing the experiences of adults with CD on a GFD. All those have been found to have an impact on the QoL of patients, as presented below [14, 17, 19].

#### 3.3.1 Acceptance and Adaptation

The diagnosis of CD brought initial relief but often transitioned into a stage of grief as patients realised the incurable nature of the disease and the need for lifelong dietary changes. The final stage was acceptance, in which individuals adjusted to a GFD:

“I just accept it. It's no different than being a diabetic. It's just what I need to do and I just do it.”

[43]

Adaptation was perceived as an ongoing learning process and an inseparable part of life, often causing frustrations:

“It was a long process of having to learn to read labels and coming to the realisation that gluten is in pretty much everything, or back then it was anyway…There was a lot of trial and error, trying new foods, throwing them out, wasting money, frustration with that. Look it took, it's taken years, it really has taken years.”

[40]

Adaptation was applied at different levels and areas of social, family, and work life. For some, this meant changing work schedules. Patients mentioned strategies used, such as batch cooking, carrying snacks, or informing those around them:

“I work shorter shifts now so my exposure is reduced. I clean obsessively. I have special soap that removes the gluten, I have my special mask. It's so strict. […]”

[44]

“I think of it like I can do this, I stay clear of gluten, no gluten at home. Explain, explain, explain to everyone ‐ 100% gluten free only. I am only gluten free.”

[44]

##### 3.3.1.1 Family Adaptation

Family adaptation emerged as a separate subtheme that involved specific dilemmas. Adaptation took various forms, such as cleaning, using different utensils, or separating areas in the kitchen. In some cases, relatives chose not to eat gluten‐containing foods in the presence of patients.

“I only cook gluten‐free now for our whole family; if they use bread, they know the drill. Butter and jam containers are labelled strictly to maintain gluten‐free; breaded chicken and pizza are the only gluten‐items allowed in the kitchen anymore and must be kept away from any other foods while out”.

[44]

“I know it's my husband's choice but at the time same time he pretty much has a GFD as well and doesn't eat those sorts of foods in front of me”.

[43]

For participants who have children, adaptation was particularly difficult:

“I find it's difficult when you've got little kids and I don't know whether to go fully gluten‐free in this house. For example, the butter dish, it's always full of breadcrumbs, so I've gone down the track of doing two butter dishes. It's really difficult. Do I have two butter dishes, two jam dishes, two honey? You know, it just gets a little bit out of control.”

[43]

Lack of support from families caused stress and put a significant strain on the participants' daily lives:

“… my family didn't really understand it [gluten free diet]… I don't think they still really understand it as in, they don't really know what gluten is or what is in gluten, they just think it's like bread or pasta…”

[25]

##### 3.3.1.2 Loss of “Normal Life”

When following a GFD, patients experienced a loss of “normal life”, often feeling socially isolated and frustrated. Some felt as if they had lost a part of themselves since starting a GFD:

“I sometimes feel I have lost myself…”

[41]

“This is so much and I cannot help but feel like I'm losing a part of myself, I've lost some of who I am. That my life is isolated and everything I do is around preventing cross contamination.”

[44]

Loss of life enjoyment due to dietary restrictions and the incurable nature of the CD were commonly reported:

“I miss that – being able to just eat anything. Not thinking about it… I sometimes hate it. I hate having to think about it. I hate having to explain that I can't eat it… you just don't want to worry about it. I just hate worrying about it. It's annoying.”

[40]

##### 3.3.1.3 Loss of Food Enjoyment

Loss of enjoyment of food was a significant concern among patients. Due to dietary restrictions, patients felt deprived of “normal” food, which changed their attitude toward eating:

“To be honest, food as a source of enjoyment is not there.”

[41]

“I've gone off food really. Food is the baddie in my life at the moment. I just eat what I have to; I've lost the enjoyment of sitting down and going out for a meal.”

[42]

#### 3.3.2 Dietary Burden

The GFD posed a constant responsibility to patients. Their thoughts were dominated by dietary aspects, which negatively impacted their daily lives.

“The constant responsibility is really hard to take.”

[41]

“You're always thinking about food. You're always cooking food”.

[42]

##### 3.3.2.1 Limited Food Availability

Patients experienced limited choices of GF foods in a variety of settings, including shops, when eating out or travelling:

“The “Free From” sections in supermarkets are disappointing, focusing mainly on biscuits and cakes, etc., the proper meal choices hardly exist, particularly for a vegetarian.”

[41]

“I still find it almost impossible to find anything suitable when in a coffee shop and often resort to eating chocolate or crisps with my coffee.”

[41]

“I cannot go on a package holiday or stay in a hotel abroad unless we self cater.”

[41]

Staff's lack of knowledge of the food's specifics contributed to the limited availability of GF products:

“…even at my workplace, you know in the canteen, when I'm trying to ask the staff member from the canteen, whether certain…let's even say, a soup of the day, whether it's full of gluten or gluten free, they are basically not aware; they don't know. So, I basically have to bring my food with myself because I can't get anything close to my workplace, or at my workplace really…”

[25]

Due to limited GF food availability, participants had to plan meals:

“I am often away from home for 12 h during the day on business and that needs careful planning to ensure I have something to eat.”

[41]

##### 3.3.2.2 Fear of Cross‐Contamination

Fear of cross‐contamination was a commonly expressed issue. Participants did their best to avoid cross‐contamination using various strategies. Nevertheless, the fear of gluten exposure was still central to their thoughts:

“I see breadcrumbs everywhere! I think of all the ways cross contamination can occur. There is a part of me that wonders if I am succumbing to some kind of collective gluten paranoia.”

[44]

“I am completely terrified to eat anywhere but my home which I share with family members that eat gluten. I'm on edge all the time, alert, watching. The fear, it's exhausting.”

[44]

Patients feared developing symptoms after cross‐contamination:

“Trouble is, if I do get an episode where I've got something [gluten] slipping through… It seems to take a little while before the symptoms disappear.”

[43]

“I'm very ill when I make mistakes, I can't let it happen.”

[42]

Fear of cross‐contamination often has led to unhealthy dietary practices, such as restricting food intake or social isolation:

“I'm worried about the crumbs, if my husband's bread is in my kitchen, I won't eat.”

[42]

“If you've got other people eating crumbs of gluten around you, that may contaminate my food. So, I actually, I tend to eat alone at work. It's very restricting. But it's worth it because if people = contamination, it's better to eat safely, and alone.”

[44]

##### 3.3.2.3 Frustrations Around the Changes in Prescription

In some areas of the UK, prescriptions for GF products were stopped or restricted. Participants affected by the changes have faced challenges, such as limited access to GF foods and financial distress:

“… since [the prescriptions were stopped] I've been buying all the stuff, it's quite difficult because it's now been, I think eighteen months at least, that I've been doing this, and over that period the availability has improved. But it can be a disaster because I go every week to two or three supermarkets, and you can get there intending to buy certain products … only to find that they've got no stock […]. The whole process is totally chaotic really …”

[25]

Although participants continued to follow a GFD, they felt the prescriptions should remain available for basic GF products, especially for vulnerable groups or large families:

“… [prescriptions] should be an option because there are people that can't afford it, you know and they're affecting their health by eating food with gluten in it. So, I think it should be an option there definitely for people, especially families with young kids …”

[25]

#### 3.3.3 Cost Burden

Both Australian and British patients with CD often mentioned the cost burden of GF products:

“The cost is usually double… Everything's a lot more expensive.”

[43]

Patients had a sense of injustice about the high prices of GF foods when comparing the prices of products on other alternative diets:

“…I get annoyed that the…having to pay through the nose through something that I can't do anything about. When you see a lot of the vegetarian stuff is same price as ordinary stuff, and you're thinking, ‘Well that's a choice that people have made; I haven't got a choice…”

[25]

The high prices of GF products forced patients to seek solutions. These included cooking and learning to shop within a budget:

“I cook myself, but I don't bake that much. So I buy the bread, and that's $6.99 [AUS$] a loaf… and you can only get about a dozen slices… I think it's too expensive. I really do and especially in country areas where we are.”

[43]

“I'm not a high income earner… I had to take [that] into consideration and to learn how to shop within my budget”.

[43]

#### 3.3.4 Socialising

Most participants reduced their social activity after starting the GFD diet:

“Socially, it's a little bit embarrassing for the people mainly because if they put [food out] and [I] say, “Sorry I can't eat it because I'm gluten‐free”… I tend not to go out much anyway.”

[43]

Participants were more likely to socialise with people who are supportive and understanding:

“My immediate family are fantastic… [the others] just seem to think that if I eat something I shouldn't then it gives me a bad stomach… It would help if there was more awareness.”

[41]

##### 3.3.4.1 Social Ignorance

The negative impact of the GFD on social life has been increased by the lack of understanding from society. It has often driven social isolation and feeling of being a “burden” to others:

“I am also met with the attitude that I shouldn't be so fussy. That I am being the difficult one. I am made to feel on lots of occasions like an outcast.”

[41]

Participants were met with disbelief and were perceived as “picky”. These reactions were often dictated by perceptions of trendy “fad” diets followed among the healthy population:

“I was once told, ‘Oh, you're one of those fussy, faddy ones, aren't you?’”

[41]

##### 3.3.4.2 Eating Out

Eating out was problematic for the patients. They feared gluten contamination, felt “different” or became the “centre of attention”:

“Eating out is always a problem and something that I don't do very often… Even if the food is gluten‐free you have no idea if it has been contaminated in the kitchen.”

[41]

“You are always ‘different’ and a bit of trouble when you go to hotels or other people's homes.”

[41]

“I hate being the centre of attention.”

[41]

Feelings of isolation and frustration were commonly experienced in food environments:

“I feel isolated. Especially at “buffet” dos.”

[41]

Patients' choices for eating out were limited to trusted places:

“… I still go out to eat but obviously you've got to go to certain places… because some of these places say they're gluten free, but then they tell you that they put the chips in the same fryers; onion rings and things like that […].”

[25]

Eating out was associated with the stress of disclosure, and some patients admitted their intentional gluten consumption in social situations:

“There may be the very odd occasion that I might have some gluten in my diet purely because I might be in a social situation… I just the pay the price a little bit later after eating gluten, if you know what I mean. So maybe in a social situation I might have a little bit of gluten very, very occasionally.”

[43]

After disclosure, participants observed gradual exclusion from social activities:

“One friend even told me she hadn't invited me to a birthday meal as she knew eating out was difficult. Whereas I understood that she was trying to save me embarrassment/discomfort, it was also quite hurtful.”

[41]

“People inviting me out to dinner inevitably wish they hadn't when they find out…generally I don't get invited back.”

[41]

#### 3.3.5 Importance of Support

Patients valued the support they received from various sources. Support facilitated adaptation, coping with the diagnosis, and eased the dietary burden.

##### 3.3.5.1 Role of Others

People close to patients living with CD played a vital role in providing daily support:

“My greatest support has been from my family and friends who make me feel normal by accepting my condition and working around it without fuss.”

[41]

##### 3.3.5.2 Role of Professional's Advice, Forums and Resources

Advice from professionals was described by patients as limited. Patients reported receiving little support or inadequate information:

“I have seen the hospital dietician twice … I knew more than she did!”

[41]

“[A nutritionist] once told me to eat Quorn, which unfortunately has wheat in it so not very good advice there!”

[41]

Communities and support groups have been reported as particularly helpful in the management of the GFD:

“My most useful and trusted source of information is via Coeliac UK.”

[41]

“The support you get from the [Coeliac] Society has been valuable, giving you so much information on all the foods and fairs.”

[41]

Due to limited support from health professionals, patients sought support and information via the Internet, reading forums, or searching for resources. For some participants, it was a source of anxiety or discouragement:

“I've also read this on the internet… Anyone else dealing with this? This gets overwhelming, I keep finding out more CC [cross contamination] dangers.”

[44]

“I often get just a little discouraged by the cheerful and enthusiastic accounts in the ‘Crossed Grain’ about how well people cope… it is not always possible to do so well.”

[41]

## 4 Discussion

Overall, the participants' experiences of GFD adherence revealed a range of challenges. For most participants, there was a period of “mourning” over the exclusion of liked foods, followed by acceptance and the process of adaptation to the GFD. Adaptations involved major lifestyle changes with rearrangement in the workplace and home, with family adaptation being a specific challenge.

Most participants expressed that a GFD had negatively impacted their QoL. The incurable nature of the condition and a need for lifelong dietary restrictions caused frustrations, feelings of loss of normal life, changed identity, and anxiety. Loss of food enjoyment was influenced by restrictions and missing “normal” foods. Patients experienced dietary burdens related to the limited availability of GF products and a feeling of constant responsibility. Despite GFD adherence, most patients feared gluten cross‐contamination. This had an impact on their social life and eating out. Many patients reduced their social activities after starting a GFD and faced widespread people's ignorance. The cost burden emerged as a separate theme and the high price of GF products was a particular challenge for those living in areas where prescriptions for GF products were restricted or stopped.

Participants recognised the importance of the support of their family and friends, as well as communities, charities, and support groups. The professional advice regarding the GFD was perceived as limited and often patients needed to do independent research. Some findings appeared helpful, while others put additional distress on patients.

Research shows that appropriately delivered professional interventions can significantly benefit patients with CD [45, 46, 47]. Costas‐Batlle et al. [45] found that a dietitian‐led coeliac service helped patients identify and minimise involuntary gluten consumption, leading to improved adherence to a GFD and reduced need for repeat endoscopies. Similarly, a study by Trott et al. [46] analysing 135 questionnaires completed by patients with CD revealed that 60% of participants found the dietitian's follow‐up “very useful”, particularly valuing the opportunity to review blood results, discuss symptoms, seek reassurance, and ask questions. Additionally, a cross‐sectional study [47] which analysed the healthcare experiences of 276 Coeliac UK members, showed that encountering more healthcare challenges related to CD management was associated with lower QoL. These findings suggest that effective healthcare professional support can enhance GFD adherence, reduce the occurrence of gluten ingestion, and improve the overall QoL for individuals with CD.

The social domain is the most negatively affected area among patients with CD on a GFD [48, 49]. Social eating is particularly problematic due to the need for disclosure in front of others. It generates the feeling of causing an inconvenience or fearing accidental gluten exposure [50]. A quantitative, UK‐based study [51] involving 146 individuals with CD revealed that 40% of participants felt “emotionally upset” due to social exclusion and embarrassment when ordering a GF option. Avoidance of disclosure when eating out is common as participants fear being rejected by society, which results in some participants' intentional gluten consumption in social situations [15, 51]. The above results echoed the findings of this meta‐synthesis, confirming a significant, negative psychological impact of GFD adherence on socialising and eating out.

Interestingly, a meta‐analysis examining the effect of GFD adherence on HRQoL in CD patients found no significant beneficial effect of the GFD [52]. Although diet adherence promoted mucosal healing and alleviated symptoms, dietary burden, and psychological distress undermined the physiological benefits. This is consistent with the findings of this meta‐synthesis, where the GFD had a mostly negative impact on the patient's psychological well‐being and overall QoL.

Loss of normal life, changed identity, and frustrations over a GFD are common in patients living with CD. Primary qualitative studies based in Europe presented similar conclusions [53, 54]. Those experiences relate to the loss of food enjoyment as well as reduced social activity, especially in the early post‐diagnosis period [16].

A dietary burden is another primary issue related to GFD adherence. A Swedish qualitative study [15] revealed similar findings with a special emphasis on a constant responsibility for a diet which meant endless checking labels, examining food, and fearing cross‐contamination. Interestingly, the participants diagnosed during childhood experienced more dilemmas around food purchase, but less concern about dietary disclosure, compared to those diagnosed as adults. In this meta‐synthesis, such conclusions could not be drawn because of a lack of information on the age at diagnosis in the examined studies. Those discrepancies highlight a need for further research in this area.

A study conducted in Canada [55] found that although the availability of GF products in North America is increasing due to the popularity of GFD, even among healthy individuals, this poses a risk of cross‐contamination due to undermining the importance of the strict dietary needs of patients with CD. Data from Europe and Australia also shows an increase in the availability of GF products, however, access to safe GF products in various environments is still an issue, especially for those living in rural areas [16, 56]. Those findings are consistent with the results of this meta‐synthesis which revealed that access to GF products is still limited in the UK and Australia, especially in the workplace or when eating out.

The support received from family and/or partners is particularly appreciated and has a significant impact on the adaptation process, as well as on reducing the dietary burden in patients living with CD [15, 57]. Patients highly value additional, nongovernmental organisations including support services and charities. Accessing face‐to‐face support services such as support groups was found to have a positive impact on the QoL of patients with CD who follow a GFD [58]. Another study, a randomised controlled trial [59] revealed positive outcomes in abdominal symptoms in patients with CD on a GFD, following the 10‐week educational programme called “Coeliac School”. Those findings confirm the results of the meta‐synthesis, raising the question of whether the support provided by health services in the UK and Australia to patients with CD is sufficient.

All findings in this meta‐synthesis supported by existing evidence reveal strong correlations between the effects of GFD adherence and an individual's psychological well‐being. With limited support available, patients' mental and physical health can be affected, leading to depression and co‐morbidities [60, 61]. This poses a query of whether health services and health professionals should consider the development of a definitive long‐term CD management strategy to offer holistic care for this group of patients, including clinical, educational, and psychological support.

## 5 Strengths and Limitations

This meta‐synthesis provided new insights into the topic of experiences of patients with CD on a GFD, being the first existing secondary qualitative research in this area.

The methodology and methods were explained in detail, which ensured the reproducibility of the paper and improved its rigour [34]. The data extraction tool and data extraction sheet were piloted, following the PRISMA guidelines [37] to meet high standards.

The use of a validated method of data synthesis—thematic synthesis by Thomas and Harden [39] and the application of data extraction tools specific to qualitative research (JBI QARI Qualitative data extraction and the extraction of the findings sheet) [38], enabled the researcher to collect relevant data, gather themes and ensured objectivity of the drawn conclusions.

The literature search involved screening four healthcare‐related databases and a reference list search enabling a broad review of existing evidence. Two studies were identified through the similar articles section on the PubMed website which is a potential limitation of the search process. The search process was illustrated in the PRISMA Flow Diagram [37] to ensure its transparency.

The researcher showed reflexivity and took the researcher's bias into account when conducting this study (see Supporting Information S1: Table S5). A validated method of data synthesis was used, and a transparent reporting system was applied. Additionally, a regular peer review with the second researcher ensured the objectivity of the findings.

According to Wray and Clarke [62], the quality of meta‐synthesis depends on the quality of analysed studies. Thus, the included articles were appraised using the CASP Tool [35] to increase the confirmability of the findings. Additionally, defining clear inclusion and exclusion criteria enhanced the credibility and dependability of the study. To ensure transparency, the reasons for study exclusion were reported. All included studies received a quality rating in the good or very good category. Four of the six studies received a “good” category in quality due to a lack of consideration of the relationship between the researcher and participants, including consideration of potential bias in data collection. The overall quality of the studies was considered “good”.

Reviewing only those papers published in English could be viewed as a limitation due to the potential omission of other relevant literature. Also, the relatively small sample of included studies may have hindered the credibility of the project as the results might not reflect the experiences of the wider population with CD on a GFD. The fact that four of the included studies were conducted in the UK and two in Australia raises questions about the extent to which the results will apply to both countries and questions the transferability and generalisability of the study to other countries.

The variables between the age, sex, and time since diagnosis among participants could be another limitation. Only two researchers specified a timeframe for participants' dietary adherence [40, 44]. The findings revealed broad experiences concerning a GFD, however, it was impossible to describe the differences in experiences of specific populations or draw conclusions based on the length of GFD adherence.

## 6 Conclusions

The study achieved its aim by revealing important insights into the experiences of adults with CD who follow a GFD in the UK and Australia. It enabled a deeper understanding of the challenges faced by this population, demonstrating significant associations between GFD adherence, psychological well‐being, and overall QoL.

### 6.1 Implications for Practice

This meta‐synthesis highlighted limitations in the support available to patients with CD. An appropriate follow‐up strategy, educational initiatives, and psychological support provided by health services and skilled professionals would help patients adjust to the diet, enabling favourable treatment outcomes and reducing the psychological burden faced by this group. This would also prevent patients from receiving incorrect suggestions and seeking support on the Internet, where not all information is accurate and evidence‐based [63, 64, 65]. Treatment of CD should therefore consist of a multidisciplinary, patient‐centred approach, considering not only the dietary and physical implications but also the psychological well‐being of patients to ensure effective, long‐term management of patients living with CD.

### 6.2 Recommendations for Future Research

This meta‐synthesis showed that the evidence on experiences of patients with CD who follow a GFD is limited in the literature from the UK and Australia, as only 6 of 286 studies met the inclusion criteria. Although there are multiple, up‐to‐date, primary qualitative studies exploring the insights of CD management in patients, they are mostly based in European countries such as Greece and Sweden.

The extensive literature search indicated that no other meta‐synthesis on this topic exists. Thus, future research could qualitatively combine evidence on the experiences of patients living with CD in different populations and more primary research in countries with limited evidence could be performed. Also, researchers should report the time since diagnosis and the timeframe for GFD adherence in individuals to allow for drawing more specific conclusions.

## Author Contributions

Anna Kowalczuk and Fiona Moor were involved in study design, screening against eligibility criteria, results' interpretation and study writeup. Database search, data extraction and data analysis were completed by Anna Kowalczuk as the primary researcher.

## Ethics Statement

Ethical approval was granted by Coventry University Ethics (reference number P146307).

## Conflicts of Interest

The authors declare no conflicts of interest.

### 1 Peer Review

The peer review history for this article is available at https://www.webofscience.com/api/gateway/wos/peer‐review/10.1111/jhn.70043.

## Supporting information

SUPPORTING INFORMATION
Supporting information can be found online in the Supporting Information section.

Supporting info.

## Data Availability

The data that supports the findings of this study are available in the Supporting Information S1 of this article.
