# Supplementary material for: A Meta‐Synthesis Exploring Daily Experiences of Adults With Coeliac Disease in Adhering to a Gluten‐Free Diet
Source: J Hum Nutr Diet. 2025 Apr 8;38(2):e70043. doi: 10.1111/jhn.70043 (PMC11977448; doi:10.1111/jhn.70043)
Supplement: Supplementary file 1 — Supporting info. [file JHN-38-0-s001.docx]

Supporting Information

**Table S1. Search Terms**

| **Research question** | How does adherence to a gluten-free diet affect the daily life experiences of adults with coeliac disease in the UK and Australia? | |
| --- | --- | --- |
|  | **Keywords**  **(Including synonyms and alternate spellings)** | **MeSH Terms / Subject Headings** |
| **Population (P)**  Adults (≥18 years) with coeliac disease in the United Kingdom or Australia | Adults; Adult; Middle aged; Elderly; Aged; Young adults  Coeliac; Celiac; Coeliac disease; Celiac disease | adult+; celiac disease |
| **Exposure (E)**  Following a gluten-free diet | Gluten free diet; Gluten Free | Diet; gluten-free |
| **Outcome (O)**  Patient experiences | Experience*; Attitude*; Perception*; Feeling*; Opinion*; Living*; Life experiences | - |
| **Study design**  Primary qualitative | Qualitative studies; Qualitative research; Qualitative methods; Qualitative; Mixed methods | - |

**Table S2 Inclusion and exclusion criteria**

| **Inclusion** | **Exclusion** |
| --- | --- |
| Coeliac disease population | Healthy population  Non-coeliac gluten sensitivity patients  Gluten intolerant patients |
| Adults ≥18 years old | Children and adolescents <18 years old |
| Gluten-free diet followed | Gluten free diet not followed |
| United Kingdom- or Australia-based | Non-United Kingdom or non-Australia based |
| Daily life experiences | No experiences reported |
| Qualitative research | Quantitative research |
| Primary studies | Secondary research |
| Written in English | Not written in English |
|  | Grey literature |


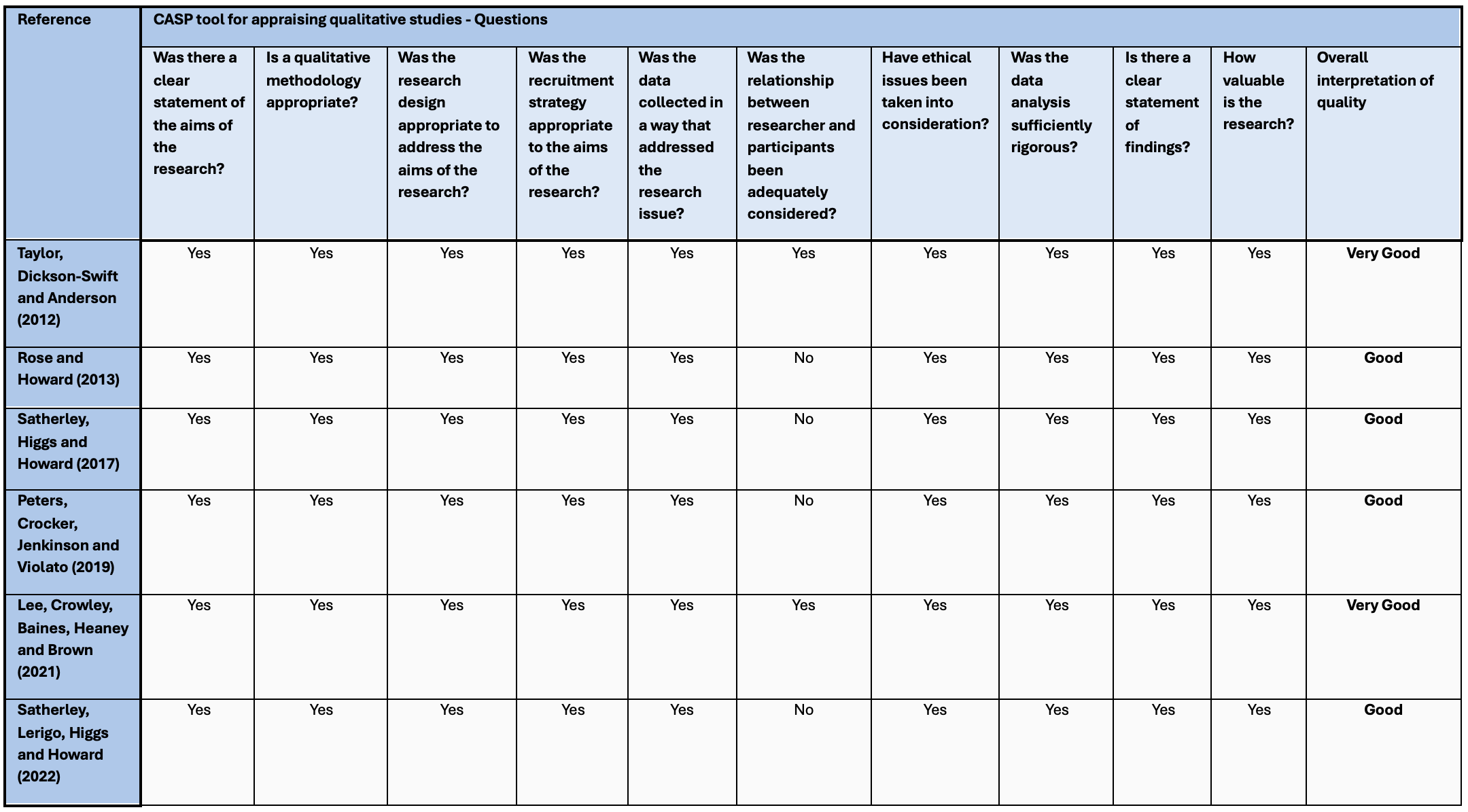
**Table S3 –** **Quality Appraisal of Included Studies - CASP Summary Table**

**Table S4 – Example search strategy – 2023.**

| **CINAHL Ultimate database search 02.06.23** | |
| --- | --- |
| **POPULATION (P)** | |
| **Key words search** | |
| S1 | **TI/AB:** Adults OR adult OR middle aged OR elderly OR aged OR young adults = 2,159,769 results |
| **CINAHL heading search** | |
| S2 | MM “adult+”  = 12,717 results |
| **Combined search** | |
| S3 | S1 OR S2  = 2,160,074 results |
| **PROBLEM (P)** | |
| **Key words search** | |
| S4 | **TI/AB:** coeliac or celiac or coeliac disease or celiac disease  = 8,403 |
| **CINAHL heading search** | |
| S5 | MH “celiac disease”  = 5,683 results |
| **Combined search** | |
| S6 | S4 OR S5  = 8,403 results |
| **EXPOSURE (E)** | |
| **Key words search** | |
| S7 | **TI/AB:** gluten free diet or gluten free  = 4,126 |
| **CINAHL heading search** | |
| S8 | MH “diet, gluten-free"  = 3,095 results |
| **Combined search** | |
| S9 | S7 OR S8  = 4,126 results |
| **OUTCOME (O)** | |
| **Key words search** | |
| S10 | **TI/AB:** experience* OR attitude* OR perception* OR feeling* OR opinion* OR living* OR life experiences  = 1,167,052 results |
| **COMBINED SEARCH (PEO)** | |
| S11 | S3 AND S6 AND S9 AND S10  = 129 results |
| S12 | **AB:** qualitative studies or qualitative research or qualitative methods  = 220,402 results |
| S13 | S11 and S12  = 18 results |
| S14 | S13  Limiters: English language  **= 18 results** |

**Table S5. Reflexivity statement**

| Reflexivity Statement – lead researcher (AK) |
| --- |
| The lead researcher (AK) participated in outpatient dietetic consultations with patients living with coeliac disease (CD) in NHS services during clinical placements from 2021-2023. During the consultations, the researcher realised the complexity of CD and its impact on the daily life of patients which developed an interest in a deeper exploration of the experiences of those patients. To minimise the risk of the researcher’s bias due to previous exposure to CD patients, peer review with the supervisor and transparency in reporting were employed. |

**Figure S1 Thematic synthesis process**

**
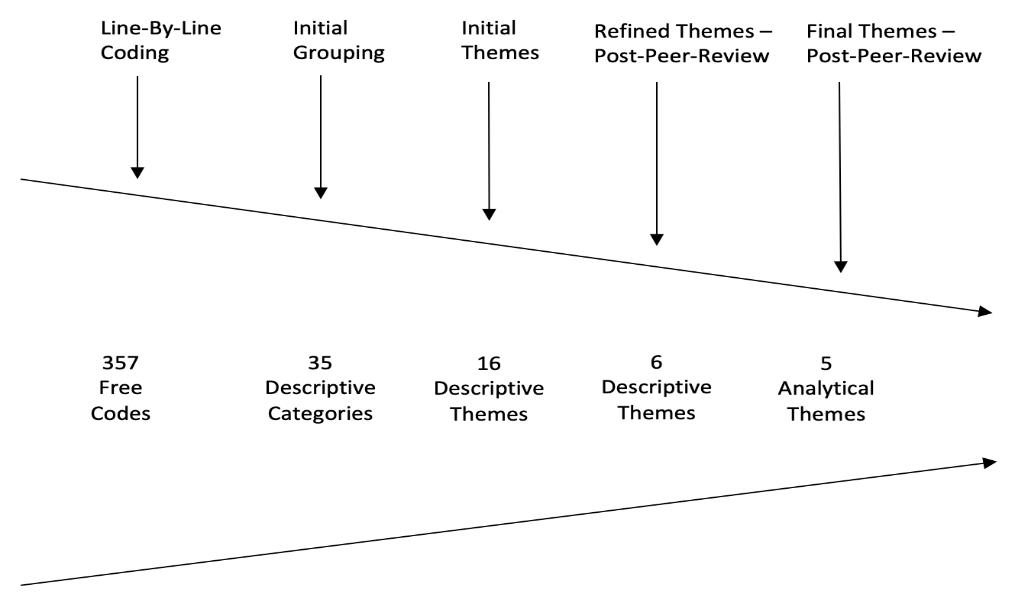
**
